# Advancements in bioinformatics and computational biology: 15th annual Polish Bioinformatic Society Symposium

**DOI:** 10.1093/bioadv/vbad187

**Published:** 2024-01-13

**Authors:** Joanna Zyla, Joanna Tobiasz, Justyna Mika, Paweł P Łabaj, Aleksandra Gruca

**Affiliations:** Department of Data Science and Engineering, Silesian University of Technology, 44-100 Gliwice, Poland; Department of Data Science and Engineering, Silesian University of Technology, 44-100 Gliwice, Poland; Department of Computer Graphics, Vision and Digital System, Silesian University of Technology, 44-100 Gliwice, Poland; Department of Data Science and Engineering, Silesian University of Technology, 44-100 Gliwice, Poland; Małopolska Centre of Biotechnology, Jagiellonian University, 30-387 Kraków, Poland; Department of Computer Networks and Systems, Silesian University of Technology, 44-100 Gliwice, Poland

## Abstract

The Polish Bioinformatic Society (PTBI) Symposium convenes annually at leading Polish Universities, and in 2023, the Silesian University of Technology hosted participants from all over the world. The 15th PTBI Symposium, spanning a 3-day duration and divided into four scientific sessions, gathered around 100 participants and centered on research related to machine learning in biomedicine, RNA structure algorithms, next-generation sequencing methods, and microbiome analysis but was not limited to only those topics. The meeting also recognized outstanding research conducted by young scientists by awarding the best poster and best talk. Finally, the awards for the best PhD, MSc, and BSc thesis in bioinformatics defended in Poland were given. This report summarizes the key highlights and outcomes of the meeting.

## 1 Introduction

The Polish Bioinformatics Society was established on 27 February 2008 and till now over 200 scientists became a member. It works to support and promote broadly defined bioinformatics research and education in Poland. The aim of the Society is to bring together and foster interaction between scientists interested in bioinformatics coming from different scientific backgrounds. The Symposium is an annual conference which is a main event organized by the Polish Bioinformatics Society and where the focus is placed to promote the research by young scientists through oral and poster presentations. Keynotes by leading researchers in the field provide further focus points for discussion at the meeting and have recently included talks by Colin de la Higuera (University of Nantes, France), Eugene Koonin (NLM/NCBI, USA), Jacek Majewski (McGill University Genome Centre, Canada), Irmtraud Margret Meyer (Max Delbruck Center For Molecular Medicine, Germany), Vilda Purutcuoglu (Middle East technical University, Turkey), Johannes Söding (Max Planck Institute for Biophysical Chemistry, Germany), Mounir Tarek (CNRS-University of Lorraine, France), or Hagen Tilgner (Weill Cornell Medicine, USA).

This year, the Symposium, which took place on 13–15 September 2023 at the Silesian University of Technology in Gliwice, hosted Djork-Arné Clevert (Pfizer, Germany), Ana Conesa (Institute for Integrative Systems Biology-SNRC, Spain), Kasthuri Venkateswaran (JPL/NASA, USA), and Joanna Sułkowska (University of Warsaw, Poland—EMBO Young Investigator). The participants also had an opportunity to listen to the invited talk on Influence of passenger mutations on expansion and extinction of cancer clones by Andrzej Polański (Silesian University of Technology, Poland) who has been awarded with honorary membership of The Polish Bioinformatics Society.

## 2 The event

### 2.1 Session I: Machine learning

The first session, chaired by Jerzy Tiuryn, was concentrated on machine learning in bioinformatics. The session started with a talk given by Witold Rudnicki entitled Novel clique-based clustering algorithm. On behalf of all authors, he presented the clustering method for studying relationships between microorganisms in the human gut. To solve the problem, the author proposed building clusters using disjoint cliques as a starting point, found by sequential greedy expansion. The obtained results were biologically meaningful and comparable with the state-of-the-art adaptive clustering. Moreover, the proposed method uses smaller hyperparameters during the analysis process compared to standard methods. The next speaker was Krzysztof Mnich, who gave a talk entitled Monte Carlo Feature Filtering: A Tale of a Tail. During the presentation, he showed that to assess whether the discovery is statistically significant using a non-parametric Monte Carlo approach (shadow variables as helper), the number of artificial variable turns is much larger than the size of the original data set. To overcome this issue, it was proposed to use a parametric approach, assuming a special form of the tail of the null distribution. It was shown that the proposed approach can give results comparable to the well-known Boruta algorithm (Kursa *et al.* 2010). Michal Marczyk gave the final talk of the session. The efficacy of breast cancer treatment varies among individuals (Marczyk *et al.* 2022), therefore patients and physicians struggle with decisions about therapy after breast surgery. In the presentation titled Developing tools to support decision-making regarding breast cancer treatment, he introduced models that help to understand how different treatments can improve survival rates for specific patients. The baseline method is based on the Fine-Gray subdistribution hazard model. However, the major development was the method of adding the influence of chemotherapy and/or hormone therapy on the patient’s survival, which allows testing any treatment regimens and can be successfully used in clinical practice.

### 2.2 Session II: Bioinformatics

Tomasz Kościółek moderated the second session, which featured five talks regarding the analysis of SNPs (single nucleotide polymorphisms), RNASeq data, and glycine formation. Firstly, Jakub Liu showed different SNPs occurrence rates in subsequent introns and exons of the human genome, indicating that the first intron and last exon have statistically more SNPs than the remaining regions in genes that consist of up to nine introns or up to five exons respectively. Next, Małgorzata Perycz presented the RNASeq analysis of human gliomas, describing the correlation between gene expression and mutations’ penetration depth for selected genes, which should be further researched. The third talk, presented by Magdalena Frąszczak concerned the analysis of long non-coding RNAs in swines’ muscle tissues, showing significant inter-individual variability and association with cell biology processes. Francisco Carrascoza gave the following talk about the mechanisms of glycine formation in the interstellar medium and on planet Earth under different conditions, indicating that fairly low energy barriers are required for glycine formation (Carrascoza *et al.* 2023). The final talk was given by Guillem Ylla, who presented their novel bioinformatic pipeline to identify, classify, and analyze both primary and secondary piRNA loci.

### 2.3 Session III: World of RNA

Jacek Błażewicz moderated the third session, which was opened with a talk by Jarosław Synak on possible regulatory mechanisms involving short RNA molecules. Three different algorithms served for verifying the model, in which short RNA molecules can bind to both complementary RNA fragments as inhibitors, and to anti-inhibitors to maintain the appropriate concentration (Synak *et al.* 2022). Next, Justyna Marek discussed the usage of deep generative models for the prediction of 3D RNA structure. This task remains challenging due to insufficient access to high-resolution structures, which limits the performance of many standard deep-learning approaches. The final talk was delivered by Norbert Dojer, who introduced the COMA (Cross-correlation Optical Map Alignment) tool dedicated to the alignment of optical mapping sequences. COMA identifies mapping locations with the double cross-correlation procedure, provides information on the locations of conflicts in alignment, and compares the results with the gold standard benchmark dataset.

### 2.4 Session IV: Low complexity regions and microbiome

Małgorzata Kotulska moderated the fourth session. Firstly, Kinga Zielińska discussed the functional aspects of the gut microbiome, highlighting the great importance of the identification of co-occurring key species. Recognition of their interactions improves the understanding of the functional profiles and hence advances the evaluation of the health status based on the gut samples. In the second talk, Sylwia Szymańska presented the language model for distinguishing publications on low complexity regions (LCRs) and their functions. She described encoding and decoding information regarding LCRs into a text embedding vector for each considered publication, which allows for more efficient analysis of the current knowledge on LCRs, unfortunately still highly unsystematized. The subsequent speech, delivered by Joanna Ziemska-Legiecka, also concerned LCRs. In particular, the co-occurrence of certain amino acids was statistically analyzed and discussed. Next, Stanisław Grodzki discussed the challenges of the effective analysis of data-independent acquisition samples and showed the novel application of Sliced Wasserstein distance. The presented approach establishes precursor-fragment relationships for ions throughout the mass-to-charge range. Igor Marchlewski gave the final talk on the study of transient tunnels to active sites. He applied and presented state-of-the-art methods of molecular dynamics simulations, capable of investigating the constantly changing properties of tunnels and providing the quantitative characterization of the ligand transport.

### 2.5 Laureates

Each year, the Polish Bioinformatic Society (PTBI) conducts the competitions for the best PhD thesis, Master thesis, and Bachelor thesis defended in Poland. Three commissions for each degree level evaluate submitted student works and select the winner. Moreover, commissions have the opportunity to indicate works to be distinguished. This year the best PhD award was given to Mateusz Rzycki for his thesis entitled Antimicrobial effect—decomposition of biological phenomena into physical approach—a theoretical model, whose parts of the thesis were published in Rzycki *et al.* (2021a,b). The award for the best master thesis went to Dominik Witczak, who presented the results of his work entitled Optimization of the GrassSV structural variant detection tool. The tool is publicly available in a GitHub repository (https://github.com/Domomod/GrassSV). Out of the bachelor thesis laureates, two of them presented their work during the meeting. First was the winner of the competition Mikołaj Żurawski who on behalf of the project team showed the results in the talk: RNApdbee 3.0: Webserver for the analysis of 3D RNA structures. The previous version of the tool was published in Zok *et al.* (2018). Next, participants had an opportunity to hear the talk given by Anna Mrukwa, who was awarded with a distinction for the best bachelor thesis entitled Deep neural autoencoder tool and unsupervised learning for scRNA-Seq data exploration.

Altogether, the PTBI Symposium 2023 featured 16 talks and 33 posters, out of which the best talk and the best poster were selected. The jury awarded Zofia Kochanska for the best talk entitled COMA—novel tool for aligning optical mapping data and Barbara Domzal for the best poster entitled Magnetstein: tool for complex mixture analysis in NMR spectroscopy. A whole list of laureates is included in Table 1.

**Table 1.** Laureates list.

Competition	Prize	Laureate	Title of thesis/talk
Best PhD thesis	First place	Mateusz Rzycki	Antimicrobial effect—decomposition of biological phenomena into physical approach—a theoretical model.
Best Master thesis	First place	Adriana Bukala	PeptiTox—a multilabel graph neural network for toxicity prediction.
	Distinction	Domninik Witczak	Optimization of the GrassSV structural variant detection tool.
Best Master thesis	First place	Mikołaj Żurawski	RNApdbee 3.0: Webserver for the analysis of 3D RNA structures.
	Distinction	Anna Mrukwa	Deep neural autoencoder tool and unsupervised learning for scRNA-Seq data exploration.
	Distinction	Radosław Jurczyk	Machine learning methods for the classification of antimicrobial peptides.
Best talk	First place	Zofia Kochańska	COMA—novel tool for aligning optical mapping data.
Best poster	First place	Barbara Domżał	Magnetstein: tool for complex mixture analysis in NMR

## 3 Conclusions

**Figure 1.**
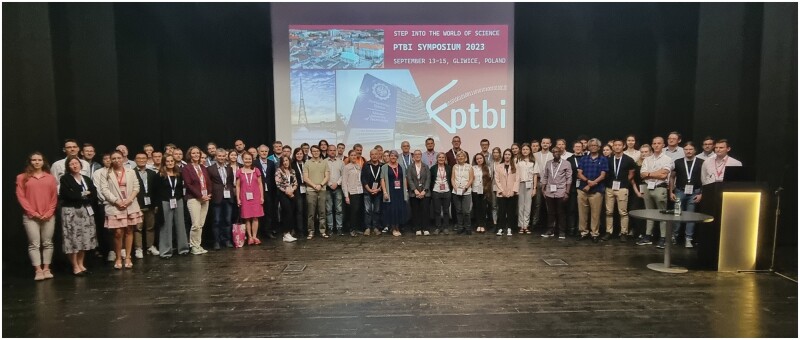
Group photo from the 15th annual Polish Bioinformatic Society Symposium.

The 15th Annual PTBI Symposium showcased cutting-edge research and developments in machine learning, bioinformatics, and their applications across various biological systems. The conference gathered experts in the field as well as young talented researchers who began their interests in bioinformatics. Participants had chances to discuss advances in their field and expand their collaborations. The group photo from the meeting is presentend in Fig. 1. Finally, the PTBI board invites the whole community for the next meeting, which will be held by Warsaw University of Technology on 11-13 September 2024.

## Data Availability

Supplementary data including a book of abstract are available at: https://www.ptbi.org.pl/website/conferences/ptbi2023/.

## References

[vbad187-B1] Carrascoza F , Lukasiak P, Nowak W et al *Ab initio* study of glycine formation in the condensed phase: carbon monoxide, formaldimine, and water are enough. Astrophys J 2023;956:140.

[vbad187-B2] Kursa MB , Jankowski A, Rudnicki WR. Boruta—a system for feature selection. Fundam Inform 2010;101:271–85.

[vbad187-B3] Marczyk M , Mrukwa A, Yau C et al; I-SPY Consortium. Treatment efficacy score—continuous residual cancer burden-based metric to compare neoadjuvant chemotherapy efficacy between randomized trial arms in breast cancer trials. Ann Oncol 2022;33:814–23. 35513244 10.1016/j.annonc.2022.04.072

[vbad187-B4] Rzycki M , Kaczorowska A, Kraszewski S et al A systematic approach: molecular dynamics study and parametrisation of gemini type cationic surfactants. Int J Mol Sci2021a;22:10939. 34681599 10.3390/ijms222010939 PMC8536075

[vbad187-B5] Rzycki M , Kraszewski S, Gładysiewicz-Kudrawiec M. Diptool—a novel numerical tool for membrane interactions analysis, applying to antimicrobial detergents and drug delivery aids. Materials2021b;14:6455. 34771982 10.3390/ma14216455 PMC8585202

[vbad187-B6] Synak J , Rybarczyk A, Blazewicz J. RNA world modeling: a comparison of two complementary approaches. Entropy 2022;24:536. 35455198 10.3390/e24040536 PMC9027272

[vbad187-B7] Zok T , Antczak M, Zurkowski M et al RNApdbee 2.0: multifunctional tool for RNA structure annotation. Nucleic Acids Res 2018;46:W30–W35. 29718468 10.1093/nar/gky314 PMC6031003

